# EST based phylogenomics of Syndermata questions monophyly of Eurotatoria

**DOI:** 10.1186/1471-2148-8-345

**Published:** 2008-12-29

**Authors:** Alexander Witek, Holger Herlyn, Achim Meyer, Louis Boell, Gregor Bucher, Thomas Hankeln

**Affiliations:** 1 Institute of Molecular Genetics, Johannes Gutenberg-University Mainz, J. J.-Becherweg 32, D-55099 Mainz, Germany; 2 Institute of Anthropology, Johannes Gutenberg-University Mainz, Colonel-Kleinmann-Weg 2, D-55099 Mainz, Germany; 3 Institute of Zoology, Johannes Gutenberg-University Mainz, Müllerweg 6, D-55099 Mainz, Germany; 4 Johann Friedrich Blumenbach Institute of Anthropology and Zoology, Georg-August-University Göttingen, J. v. Liebig-Weg 11, D-37077 Göttingen, Germany

## Abstract

**Background:**

The metazoan taxon Syndermata comprising Rotifera (in the classical sense of Monogononta+Bdelloidea+Seisonidea) and Acanthocephala has raised several hypotheses connected to the phylogeny of these animal groups and the included subtaxa. While the monophyletic origin of Syndermata and Acanthocephala is well established based on morphological and molecular data, the phylogenetic position of Syndermata within Spiralia, the monophyletic origin of Monogononta, Bdelloidea, and Seisonidea and the acanthocephalan sister group are still a matter of debate. The comparison of the alternative hypotheses suggests that testing the phylogenetic validity of Eurotatoria (Monogononta+Bdelloidea) is the key to unravel the phylogenetic relations within Syndermata. The syndermatan phylogeny in turn is a prerequisite for reconstructing the evolution of the acanthocephalan endoparasitism.

**Results:**

Here we present our results from a phylogenomic approach studying i) the phylogenetic position of Syndermata within Spiralia, ii) the monophyletic origin of monogononts and bdelloids and iii) the phylogenetic relations of the latter two taxa to acanthocephalans. For this analysis we have generated EST libraries of *Pomphorhynchus laevis*, *Echinorhynchus truttae *(Acanthocephala) and *Brachionus plicatilis *(Monogononta). By extending these data with database entries of *B. plicatilis*, *Philodina roseola *(Bdelloidea) and 25 additional metazoan species, we conducted phylogenetic reconstructions based on 79 ribosomal proteins using maximum likelihood and bayesian approaches. Our findings suggest that the phylogenetic position of Syndermata within Spiralia is close to Platyhelminthes, that Eurotatoria are not monophyletic and that bdelloids are more closely related to acanthocephalans than monogononts.

**Conclusion:**

Mapping morphological character evolution onto molecular phylogeny suggests the (partial or complete) reduction of the corona and the emergence of a retractable anterior end (rostrum, proboscis) before the separation of Acanthocephala. In particular, the evolution of a rostrum might have been a key event leading to the later evolution of the acanthocephalan endoparasitism, given the enormous relevance of the proboscis for anchoring of the adults to the definitive hosts' intestinal wall.

## Background

**Figure 1:**
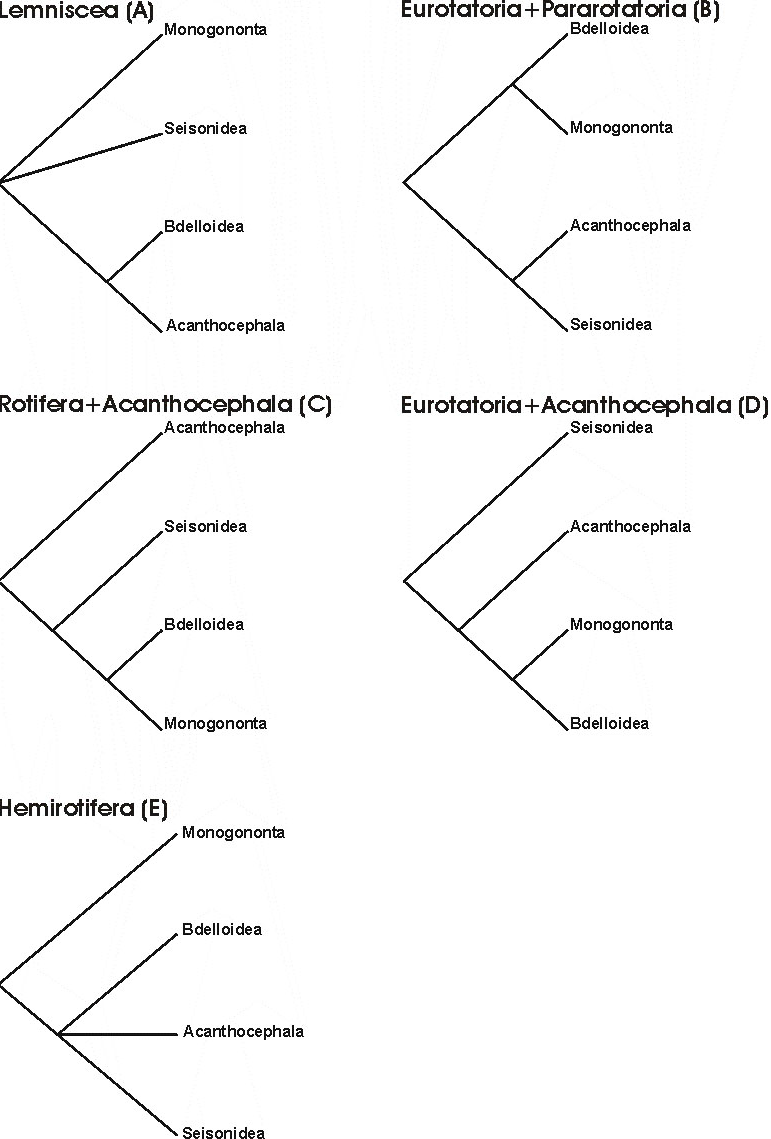
**Competing phylogenetic hypotheses amongst Syndermata**. Cladograms reflecting the competing hypotheses on the phylogenetic relations among Monogononta, Bdelloidea, Acanthocephala and Seisonidea. A Lemniscea hypothesis [23]. B Eurotatoria+Pararotatoria hypothesis [5,9,12]. C Rotifera+Acanthocephala [8,26]. D Eurotatoria+Acanthocephala [28]. E Hemirotifera [29].

The animal taxon Rotifera comprises free-living and commensalic microscopic species of aquatic habitats that are traditionally grouped into the three subtaxa Bdelloidea, Monogononta and Seisonidea [1-3]. Bdelloids (about 460 species) inhabit freshwater, are capable of anhydrobiosis and reproduce strictly by parthenogenesis. Monogononts (about 1,570 species) live in limnic, brackish and marine waters and have a lifecycle with alternating phases of parthenogenetic and sexual reproduction. Thirdly, at least two of the hitherto three described species belonging to Seisonidea are epibionts on marine crustaceans of the genus *Nebalia *[3,4]. Though Bdelloidea, Monogononta and Seisonidea are subsumed as Rotifera or Rotatoria, the eponymous rotatory organ or corona, a seemingly rotating assembly of cilia at the anterior end of the animal, is absent in Seisonidea. For this and other reasons Bdelloidea and Monogononta are often regarded as sistergroups of a taxon named Eurotatoria [2,5,6]. In contrast to Bdelloidea, Monogononta and Seisonidea, the Acanthocephala are obligatory endoparasites with a complicated lifecycle. Their definite hosts are vertebrates, while their intermediate hosts are insects, chilopods and crustaceans (e.g., Meyer [7]). Along with the endoparasitic life cycle, the acanthocephalan subtaxa share a plethora of derived morphological characters (e.g., [8-11]) so that the monophyly of Acanthocephala as a whole has never been debated. Moreover, the grouping of Acanthocephala, Bdelloidea, Monogononta and Seisonidea into the taxon Syndermata is widely accepted due to special features in epidermal and sperm ultrastructure (e.g., syncytial epidermis, spermatozoon with anteriorly inserted cilium; see [5,8,9,12,13]), as well as congruent results from molecular approaches [14-20]. It is further undisputed that Syndermata are part of a more comprehensive monophylum called Gnathifera [9,21,22]. On the other hand, the phylogenetic position of Syndermata beyond Gnathifera as well as the relationships among the syndermatan subtaxa Acanthocephala, Bdelloidea, Monogononta and Seisonidea are still unresolved. So far, five competing hypotheses on the internal phylogeny of Syndermata have been suggested (Fig. 1A–E). The Lemniscea hypothesis goes back to Lorenzen [23] and favors a sister group relationship of bdelloids and acanthocephalans, with the Monogononta and Seisonidea placed basally to the Lemniscea (Fig. 1A). Morphological evidence for such grouping has been inferred from two lateral intrusions in the neck region and a retractable anterior body section in Acanthocephala and Bdelloidea [23]. The Lemniscea hypothesis received additional support from 16S rRNA, 18S rRNA, 28S rRNA, cytochrome *c *oxidase subunit 1 (*cox *1) and histone *H3 *data [14-17,19]. The second hypothesis suggests a sistergroup relationship of Monogononta and Bdelloidea (Eurotatoria) and of Seisonidea and Acanthocephala (Pararotatoria) and is herein called Eurotatoria+Pararotatoria hypothesis (Fig. 1B). Besides presumed eurotatorian apomorphies such as the already mentioned corona, the Eurotatoria+Pararotatoria hypothesis is based on ultrastructural peculiarities that have been interpreted as synapomorphies of Seisonidea and Acanthocepahala (spermatozoa with "dense bodies" and epidermis with special filaments [5,9,12]). Additional support for the monophyly of Pararotatoria came from partial 18S rRNA data [24] as well as from a combined dataset of 18S rRNA sequences, heat shock gene sequences (*hsp82*), and morphological characters [25]. The third hypothesis reflects the classical view of monophyletic Rotifera (Monogononta+Bdelloidea+Seisonidea) and Eurotatoria (Monogononta+Bdelloidea) and proposes Acanthocephala as the sistergroup of Rotifera ("classical Rotifera+Acanthocephala hypothesis", see Fig. 1C). This classical concept has been formulated based on specific features of toe morphology, sensory and masticatory apparatus in Rotifera and Eurotatoria, respectively [13,26], and was supported by 18S rRNA data [27]. The fourth hypothesis has been proposed on the basis of *hsp82 *sequences, and groups Acanthocephala and Eurotatoria with exclusion of Seisonidea [28] ("Eurotatoria+Acanthocephala", see Fig. 1D). Underlying the fourth hypothesis, the absence of acrosomal structures might represent a synapomorphy of Eurotatoria and Acanthocephala [21]. According to the fifth hypothesis, Bdelloidea, Seisonidea, and Acanthocephala form a monophylum for which the name Hemirotifera has been proposed (Fig. 1E). The Hemirotifera hypothesis has been inferred from a combined dataset of molecular (18S rRNA, 28S rRNA, histone *H3*, *cox 1*) and morphological characters [29]. This survey of competing hypotheses demonstrates that the question of phylogenetic relationships within Syndermata and therewith of the evolution of the acanthocephalan endoparasitism is closely connected to the more basal question of monophyly of Eurotatoria.

In the present study we analyse the phylogenetic position of Syndermata within the spiralian clade as well as the phylogenetic relations among the syndermatan subtaxa. We particularly focus on the question whether Eurotatoria are monophyletic and – if not – whether bdelloids or monogononts are more closely related to acanthocephalans. As ribosomal proteins are favorable tools for metazoan molecular phylogenetic analyses [18,30-32] and easy to obtain from EST libraries, we compiled a phylogenomic dataset comprising 79 ribosomal proteins. To this end, we generated EST libraries for one monogonont (*B. plicatilis*) and two acanthocephalans (*P. laevis and E. truttae*; both Echinorhynchida) and sequenced 1,000–2,000 ESTs per library. The new sequences were complemented with ortholog data from public databases for the monogonont *B. plicatilis*, the bdelloid *P. roseola *and 25 additional metazoan taxa. Data of Seisonidea have not been included in the present analysis as it is extremely difficult to obtain sufficient material for the preparation of a cDNA library. As a beneficial side effect, the present tree reconstruction cannot be disturbed by the observed long branch leading to representatives of Seisonidea (see, e.g., [19,24]).

## Results

### Sequence analyses and ribosomal protein alignment

EST sequencing was performed for three syndermatan species and complemented by sequences from public databases (Tab. 1). A dataset containing the coding sequences of 79 ribosomal proteins was extracted, and derived amino acid sequences were concatenated. After cleaning the raw data from ambiguously aligned positions, the final alignment had a length of 11,276 amino acids. Fifteen to 29 species were sampled per protein type, resulting in a taxon coverage ranging from 36 to 100% outside Syndermata (see Additional files 1 and 2 for the complete matrix of taxa and ribosomal proteins used and the amino acid coverage of each ribosomal protein), and 28 to 89% within Syndermata, compared to the vertebrate reference. Given the length of the complete dataset the minimum coverage within the syndermatan sample (see *E. truttae *in table 2) still represents 3,204 amino acid positions which is a considerable increase in data compared to previous analyses of syndermatan phylogeny (e.g., [17,24]). The total coverage of Acanthocephala, however, is much higher than suggested by the *E. truttae *data alone, due to the additional 66% sequence coverage in *P. laevis *(table 2).

**Table 1:** List of the syndermatan species for which new data have been collected in the present analysis

Species	Taxon	Origin	# EST	# RP
*Pomphorhynchus laevis*	Acanthocephala (Palaeacanthocephala)	Gravel pit at Gimbsheim, Germany (from host *Barbus fluviatilis*)	2.207	65
*Echinorhynchus truttae*	Acanthocephala (Palaeacanthocephala)	River Leine at Göttingen, Germany (from host *Salmo trutta fario*)	1.440	23
*Brachionus plicatilis*	Monogononta	Lab culture + public data	2.000	16 (28)
*Philodina roseola*	Bdelloidea	Public data	None	0 (72)

For *Brachionus *and *Philodina *the number of ribosomal proteins found in our EST datasets is shown outside brackets, the number in the combined datasets is within brackets. A complete list of taxa and ribosomal proteins used in this analysis can be found in Additional file 2.

**Table 2:** Syndermatan coverage in the dataset

Species	Taxon	# amino acids	% of coverage
*Pomphorhynchus laevis*	Acanthocephala	7,430	65.89
*Echinorhynchus truttae*	Acanthocephala	3,204	28.41
*Brachionus plicatilis*	Monogononta	4,255	37.74
*Philodina roseola*	Bdelloidea	10,005	88.73

Absolute number of amino acid positions used and relative coverage of the sequence alignment for the four syndermatan representatives analyzed in the present study.

**Figure 2:**
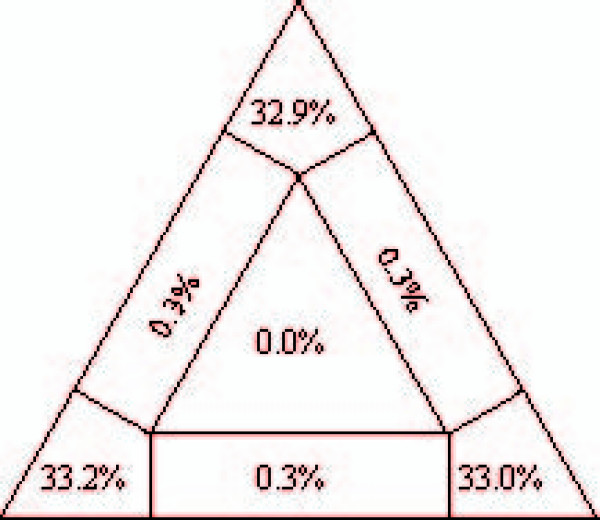
**Likelihood mapping of the concatenated alignment**. Results from likelihood mapping of the concatenated ribosomal protein sequences from 29 species analysed in the present study. Note that 99.1% of all quartets were fully resolved and none of the quartets produced a star-like tree.

Likelihood mapping analysis determined a strong phylogenetic signal in the data. In detail, 99.1% of the quartets were fully resolved and none of the quartet-trees showed a star-like topology (Fig. 2). Furthermore, we found no evidence for horizontal gene transfer in the bdelloid dataset, applying the test statistics proposed by Gladyshev et al. [33]: As to be expected for ribosomal genes, the so-called Alien Index was < 0 for each protein. We therefore consider our dataset a sound basis for assessing the phylogenetic position of Syndermata within Spiralia, and for answering the question of eurotatorian monophyly.

### Phylogenetic reconstruction

**Figure 3:**
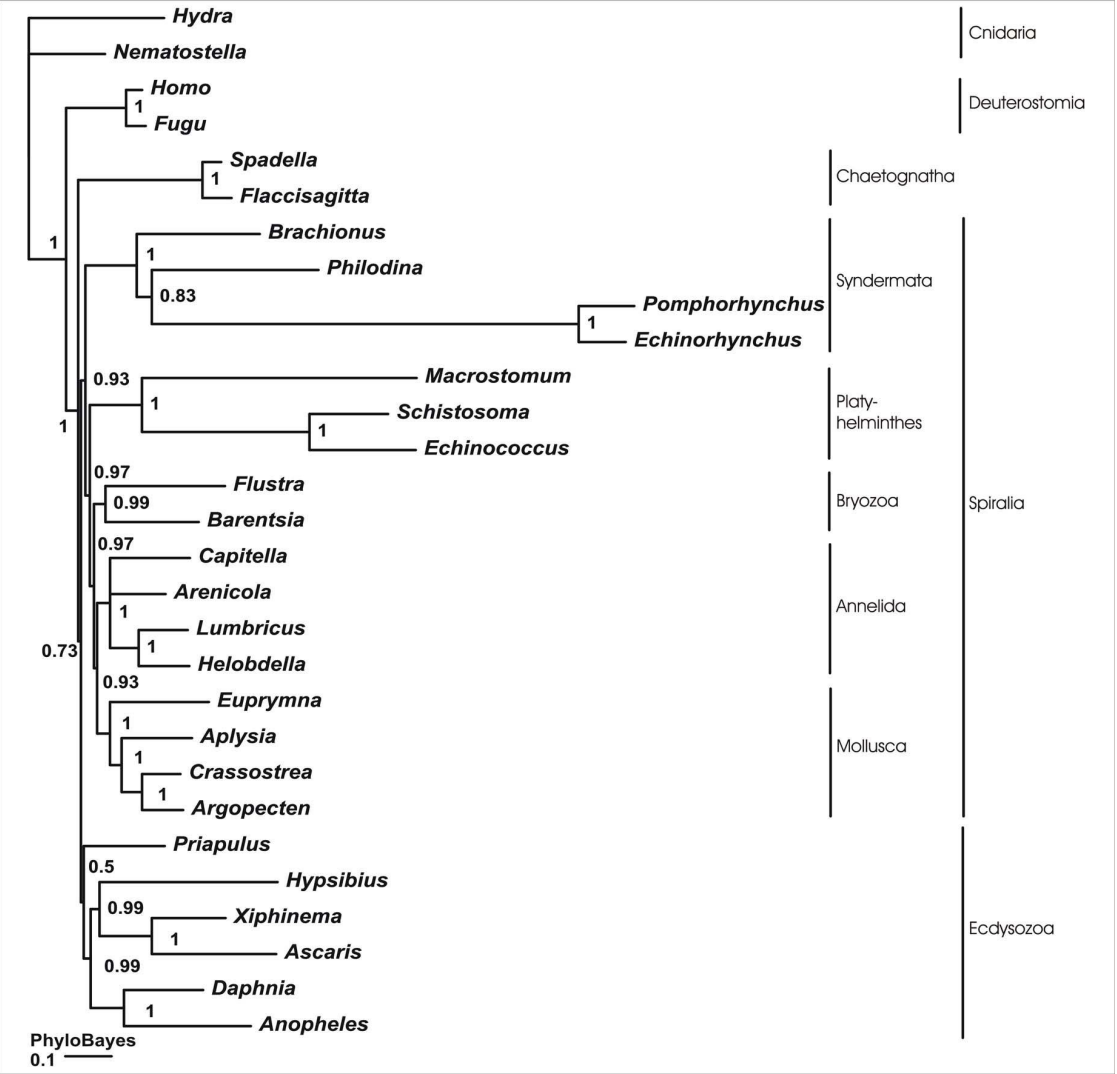
**Phylogenetic tree reconstruction using bayesian inference (Phylobayes).** Numbers at internal nodes represent posterior probabilities. Syndermata are shown as a basal spiralian taxon. Moreover, Eurotatoria appear paraphyletic, with Bdelloidea being more closely related to Acanthocephala than to Monogononta.

**Figure 4:**
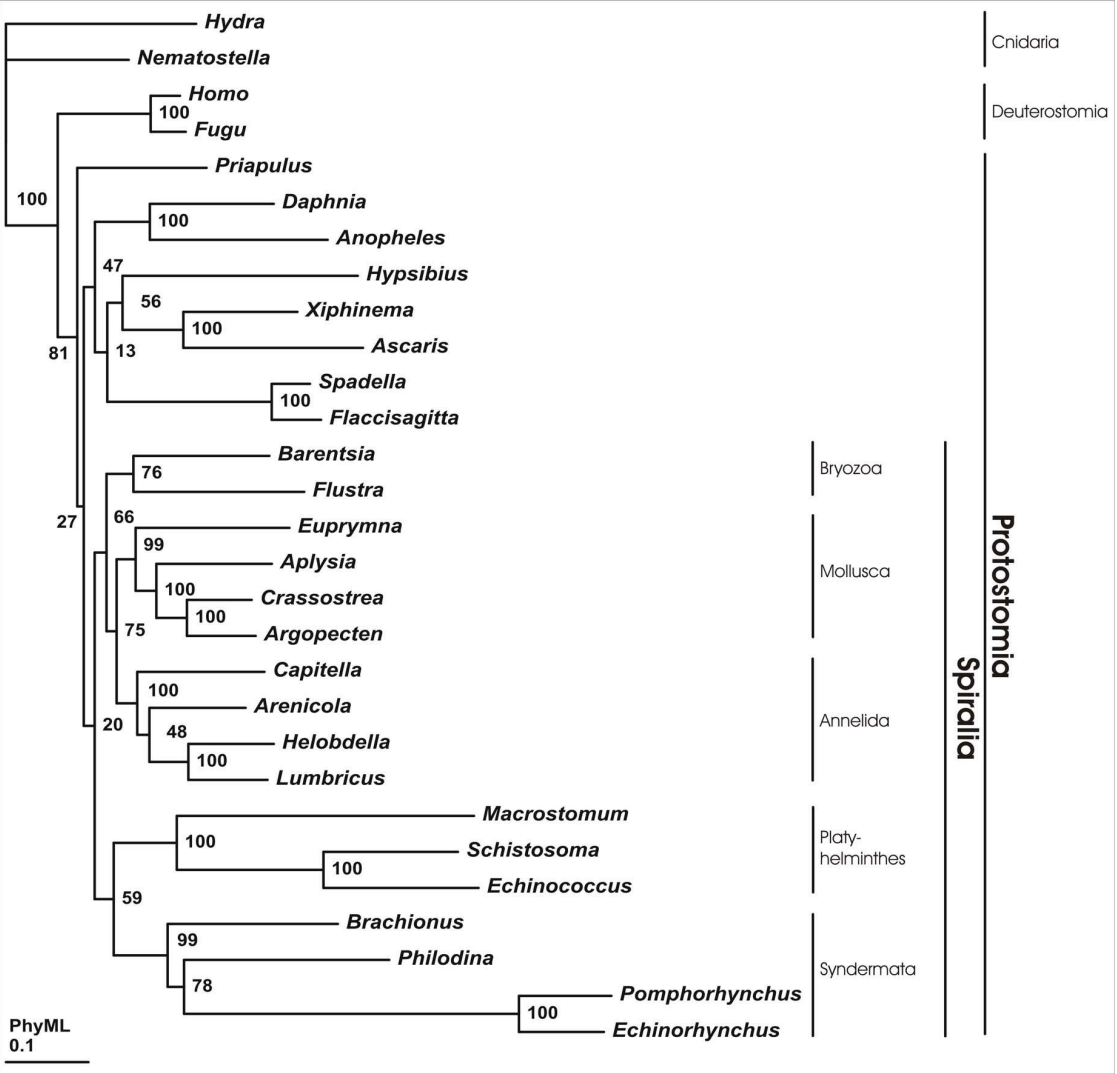
**Phylogenetic tree reconstruction using maximum likelihood (PhyML).** Numbers at internal nodes represent bootstrap values. Syndermata are shown as a spiralian taxon, with a sistergroup relationship to Platyhelminthes. Moreover, Eurotatoria appear paraphyletic, with Bdelloidea being more closely related to Acanthocephala than to Monogononta.

**Figure 5:**
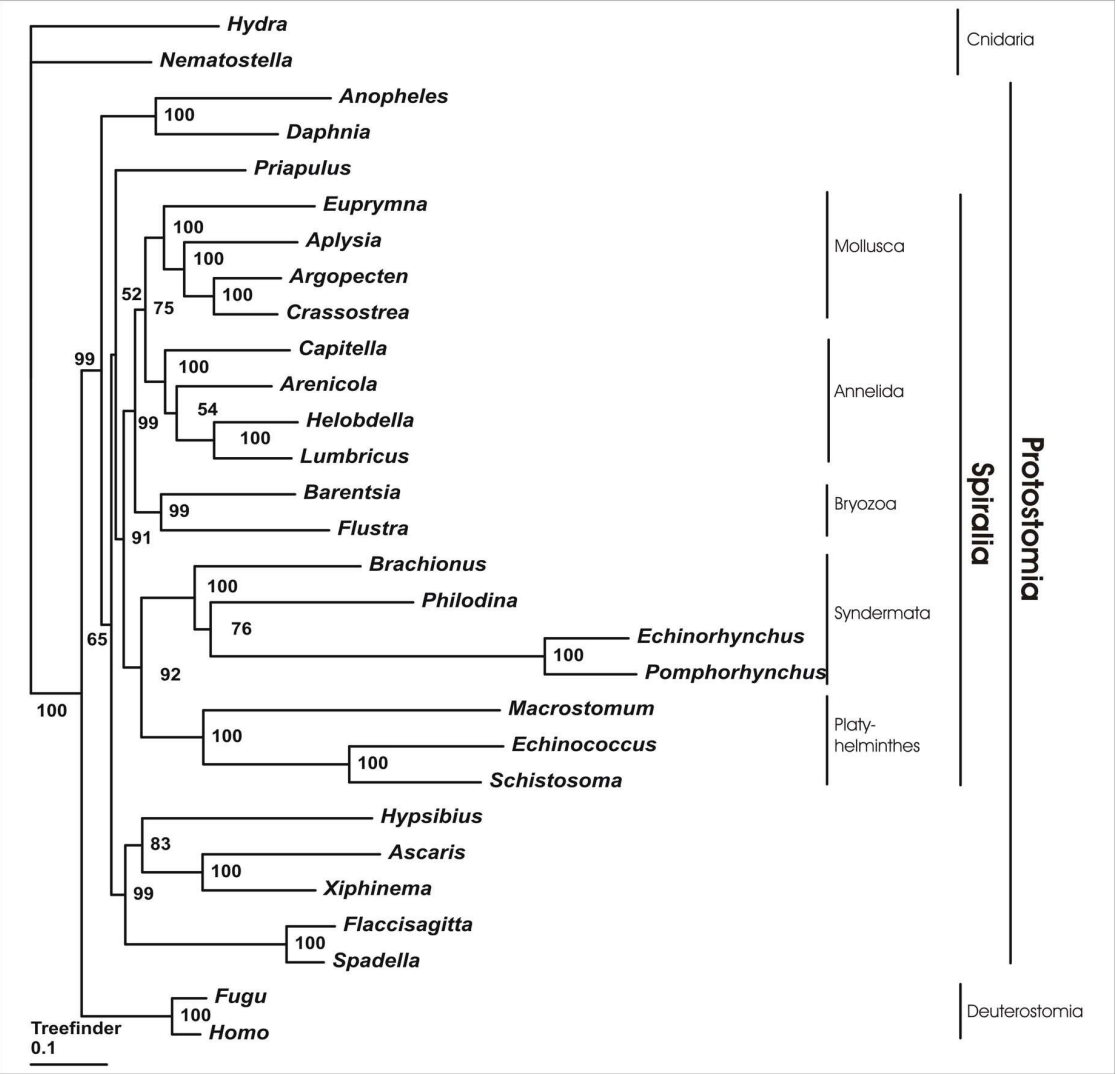
**Phylogenetic tree reconstruction using maximum likelihood (Treefinder).** Numbers at internal nodes represent expected likelihood weights. Syndermata are shown as a spiralian taxon, with a sistergroup relationship to Platyhelminthes. Moreover, Eurotatoria appear paraphyletic, with Bdelloidea being more closely related to Acanthocephala than to Monogononta.

Maximum likelihood and bayesian phylogenetic inference consistently support a monophyletic origin of Spiralia, although with partly moderate support values (PhyML < 50; Treefinder: 91; PhyloBayes: 0.93) (Fig. 3, 4, 5). Interestingly, only the PhyloBayes tree depicts the widely accepted monophylum Ecdysozoa (e.g., [16]) so that bayesian inference might provide more reliable results than the maximum likelihood approaches employed, given the present data (Fig. 3). Irrespective of this detail, all three tree reconstruction methods yield maximum support for a monophyletic origin of *B. plicatilis*, *P. roseola*, *E. truttae *and *P. laevis *and, thus, for the monophyletic origin of the four syndermatan species covered by the present dataset (PhyML: 100; Treefinder: 100, PhyloBayes: 1.00). Within the spiralian clade, Syndermata either group with Platyhelminthes (PhyML: 59; Treefinder: 92) (Fig. 4 and 5) or with a clade comprising Platyhelminthes, Bryozoa, Mollusca and Annelida (PhyloBayes: 0.93) (Fig. 3). Remarkably, none of the tree reconstruction methods supports a sister group relationship of Monogononta and Bdelloidea. Instead, Bdelloidea consistently appear more closely related to Acanthocephala than to Monogononta (PhyML: 78; Treefinder: 76; PhyloBayes: 0.83). The paraphyly of Eurotatoria is further corroborated by results from hypothesis testing. Thereafter, a grouping of Bdelloidea+Acanthocephala is much more likely than the alternatives Monogononta+Bdelloidea and Monogononta+Acanthocephala (Tab. 3). Final evidence for the robustness of the present analysis comes from testing for the effect of missing data in the full-length dataset on the results of tree reconstruction [41]. Thus, the internal syndermatan phylogeny did not change when tree reconstruction was carried out on the basis of a shorter dataset (24 ribosomal proteins, 3,535 amino acid positions) in which all ribosomal protein sequences had orthologs in acanthocephalans, bdelloids and monogononts. Support for a grouping of Bdelloidea and Acanthocephala was even higher when underlying this shorter dataset (PhyML: 83; Treefinder: 85; PhyloBayes: 0.92). Taken together our data suggest i) Syndermata being Spiralia with a close phylogenetic relation to Platyhelminthes and ii) the paraphyly of Eurotatoria, with iii) Bdelloidea being more closely related to Acanthocephala than to Monogononta.

**Table 3:** Results from hypotheses testing

Hypothesis	ELW
Bdelloidea + Acanthocephala	1
Bdelloidea + Monogononta (Eurotatoria)	0
Monogononta + Acanthocephala	0

Results from hypotheses testing based on the three alternative Acanthocephala-sistergroup relationships covered by the present approach using the Expected Likelihood Weight (ELW) test.

## Discussion

The phylogenetic position of Syndermata within Spiralia has been described previously based on molecular data such as 18S rRNA and 16SrRNA [15], 28S rRNA and 18S rRNA [20], ribosomal proteins [18] and on morphological characters like spiral cleavage, filiform sperm without accessory centriole and the subepidermal cerebral ganglion [5]. Likewise, a close phylogenetic relationship of Syndermata and Platyhelminthes within the spiralian clade agrees well with results from previous molecular and morphological approaches on metazoan phylogeny (e.g., [15,18,20,34]). On the other hand, conflicting results from the present tree reconstructions indicate that even the analysis of up to 79 ribosomal proteins from up to 29 species cannot settle the question of the definite phylogenetic relationship of Syndermata and Platyhelminthes. In agreement with Dunn et al. [34] we recommend an enlarged taxon sampling and the incorporation of more closely related groups such as gnathostomulids and micrognathozoans for resolving the question of the sistergroup relationships of Syndermata and Gnathifera, respectively.

In contrast to the still contradictory results regarding the phylogenetic position of Syndermata within Spiralia, our tree reconstructions consistently depict Bdelloidea as more closely related to Acanthocephala than to Monogononta, though with partly moderate support (Fig. 3, 4 and 5). As the moderate support values have been calculated on the basis of the full-length dataset, they might be due to missing data in one or more of the syndermatan lineages sampled. This is at least suggested by the higher statistical support for a clade Bdelloidea+Acanthocephala that could be inferred from the shorter dataset without these missing data (PhyML: 83; Treefinder: 85; PhyloBayes: 0.92). Regardless of differences in the support, analyses of both datasets lead to the same topology among the syndermatan representatives, whichever algorithm was employed. We take this high nodal stability (reflected also by unambiguous results from hypotheses testing) as evidence for reliability of the found grouping of Acanthocephala and Bdelloidea (see [35] for a discussion of nodal stability in the formulation of phylogenetic hypothesis). The future incorporation of EST data from Gnathostomulida and Micrognathozoa, and especially their use as outgroup, will be necessary to yield improved nodal support for the implicit paraphyly of Eurotatoria.

As another note of caution, one has to be aware that the present results as well as consistent results from more limited datasets [24-26] could in principle be influenced by an acceleration of sequence evolution on the branch of the only monogonont sampled, i.e. *B. plicatilis*. It is thus conceivable that a deviating mode of sequence evolution in *B. plicatilis *(as described for *hsp82 *[28]) triggered an attraction of the bdelloid and acanthocephalan branches. On the other hand, this is not very likely as the tree reconstruction methods employed herein (maximum likelihood and bayesian inference) are relatively robust to long-branch attraction. Moreover, Eurotatoria appeared paraphyletic in previous analyses comprising several monogononts [17,19] which cannot be explained by long-branch attraction due to an accelerated sequence evolution in *B. plicatilis*. We therefore do not believe that a faster sequence evolution along the *B. plicatilis *branch is causative for the found clustering of Bdelloidea and Acanthocephala.

The present evidence for a paraphyly of Eurotatoria is in apparent conflict with three out of the five competing hypotheses on the intra-syndermatan phylogeny, i.e. the Eurotatoria+Pararotatoria hypothesis (Fig. 1B), the classical Rotifera+Acanthocephala hypothesis (Fig. 1C), and the Eurotatoria+Acanthocephala hypothesis (Fig. 1D). At first sight, the observed grouping of Bdelloidea and Acanthocephala (under exclusion of Monogononta) rather supports the predictions of the Lemniscea hypothesis of Lorenzen ([23]). However, considering previous evidence from morphological [5,9,12] and molecular data [24] as well as from approaches combining both types of data [25,29], it is still possible that Seisonidea represent the true acanthocephalan sister taxon. However, it cannot be ruled out that Seisonidea are the sistergroup of Bdelloidea, Pararotatoria or Monogononta+Acanthocephala (see single trees in [19,25]).

Given the uncertain phylogenetic position of Seisonidea within Syndermata, one has to be cautious when inferring the evolution of morphological characters. On the other hand, the well supported closer relation of Bdelloidea to Acanthocephala, with exclusion of Monogononta ([19], present study), allows for some conclusions regarding the evolution of morphological characters that are not bound to the position of Seisonidea within Syndermata. It is thus very likely that the rotatory organ or corona underwent a (partial or total) reduction before the separation of Acanthocephala. A likewise reduction of a newly emerged character (wings) has for example been described in stick insects (Phasmatodea; [36]). Therefore the reduction of the rotatory organ only a few splits after its emergence at the base or within the syndermatan tree is not as unlikely as it might appear at first sight. Another implication of the grouping of Acanthocephala and Bdelloidea is that a retractable anterior end – whether in the shape of a rostrum in Bdelloidea or as a hooked proboscis in Acanthocephala – probably evolved before the separation of the acanthocephalan stem lineage as well. The reduction of the corona as well as the evolution of a retractable anterior end can easily be explained by different life-styles and patterns of locomotion in the syndermatan subtaxa: free living/free swimming in Monogononta; leech-like creeping/free living in Bdelloidea; leech-like creeping/epibiontic in Seisonidea; reduced motility/endoparasitic in Acanthocephala (see also [24,37-39]). Particularly the early evolution of a retractable anterior end might have represented a key event leading to the later evolution of the acanthocephalan endoparasitism, given the crucial role of the proboscis in the anchoring of adult acanthocephalans to the definitive hosts' intestinal wall [40].

## Conclusion

Based on a dataset comprising sequences from up to 79 ribosomal proteins of up to 29 species, we provide evidence for the paraphyly of Eurotatoria. Irrespective of the tree reconstruction method and dataset used (and additionally supported by hypothesis testing) we found Bdelloidea to be more closely related to Acanthocephala than to Monogononta. Although data for Seisonidea have not been included in the dataset, the present findings allow for the rejection of three (Eurotatoria+Pararotatoria, Eurotatoria+Seisonidea, Eurotatoria+Acanthocephala) out of the presently five competing hypothesis regarding the phylogeny within Syndermata. On the other hand, additional data are needed to determine the actual acanthocephalan sistergroup (Seisonidea or Bdelloidea). Irrespective of these limitations it is very likely that a (partial or complete) reduction of the rotatory organ or corona occurred before the separation of Acanthocephala. Likewise, a retractable anterior end most likely emerged before the separation of the acanthocephalan stem lineage. Considering the importance of the proboscis for the attachment of acanthocephalans to the definite host's intestinal wall, the latter step can be regarded as a key event towards the evolution of acanthocephalan endoparasitism.

## Methods

### Isolation of RNA and cDNA library construction

Total RNA was extracted from frozen pooled specimen using column-based methods (Qiagen RNeasy Plant Mini Kit, Qiagen, Hilden, Germany). Quality of RNA was visually checked on agarose gels and mRNA was subsequently captured using the NucleoTrap mRNA kit (Macherey-Nagel, Düren, Germany) for *B. plicatilis *and the polyATract mRNA Isolation System III (Promega, Mannheim, Germany) for *P. laevis *and *E. truttae*. cDNA libraries were constructed at the Max Planck Institute for Molecular Genetics in Berlin (*P. laevis*) and the Institute of Molecular Genetics, University of Mainz (*E. truttae*, *B. plicatilis*) by primer extension (*P. laevis*, *B. plicatilis*) or LD-PCR (*E. truttae*), size fractionation and directional cloning applying the Creator SMART cDNA Libraries Kit (Clontech, Heidelberg, Germany) with the vectors pDNR-LIB or a modified pSPORT [41]. Clones containing cDNA inserts were sequenced from the 5' end on ABI 3730 capillary sequencer systems using BigDye chemistry (Applied Biosystems, Darmstadt, Germany).

### EST processing

EST processing for *P. laevis *was accomplished at the Center for Integrative Bioinformatics in Vienna. Sequence chromatograms were first base-called and evaluated using the Phred application [42]. Vector, adaptor, poly-A tract and bacterial sequences were removed employing the software tools Lucy http://www.tigr.org, SeqClean http://compbio.dfci.harvard.edu/tgi/software, and CrossMatch http://www.phrap.org, respectively. Clustering and assembly of the clipped sequences was performed using the TIGCL program package http://compbio.dfci.harvard.edu/tgi/software by performing pairwise comparisons (MGIBlast) and a subsequent clustering step (CAP3). Low quality regions were then removed by Lucy. Finally, contigs were tentatively annotated by aligning them pairwise with the 25 best hits retrieved from NCBI's non-redundant protein database using the BlastX algorithm http://www.ncbi.nlm.nih.gov. Alignment and computation of the resulting match scores, on which the annotation was based, were conducted by GeneWise [43] in order to account for frame shift errors.

ESTs for *E. truttae *and *B. plicatilis *were processed semi-automatically: removal of vector parts, polyA tails and bad quality sequence from sequence traces was performed by the SeqMan option of the DNASTAR program suite (Lasergene). Overlapping EST sequences were clustered using SeqMan (Lasergene). For *B. plicatilis *publicly available data from dbEST and the trace archives were included into the clustering process, and public data for *Philodina roseola *was clustered the same way. For annotation, EST cluster consensus sequences and EST singletons were subjected to BLASTX comparison against the SWISS-PROT protein database at NCBI http://www.ncbi.nlm.nih.gov/, using a BLAST client tool (Blastcl3, Blast software package, NCBI) setting the cut-off to 1*e^-10^. The EST data used in our analyses have been deposited in Genbank under the accession numbers [GenBank: AM849482 – AM849546 (*P. laevis*), AM980962 – AM980984 (*E. truttae*) and AM980946 – AM980961 (*B. plicatilis*)].

### Sequence analysis and ribosomal proteins alignment

Ribosomal protein sequences were extracted from the newly obtained and publicly available EST data by their annotation. EST sequence contigs were checked for assembly errors by visual inspection and by comparison with corresponding sequences of related taxa, and translated into amino acid sequences. Gladyshev et al. [33] recently reported evidence for gene aquisition by horizontal gene transfer in two bdelloid species. Although it is unlikely that ribosomal proteins are subject to horizontal gene transfer, but as a precaution, we checked whether our data are influenced by horizontal gene transfer or not. Therefore we performed Blastp searches with our amino acid sequences and calculated the 'Alien Index' as introduced by Gladyshev et al. [33]. This index represents a measure of the orders of magnitude by which the BLAST E-value for the best metazoan hit differs from that for the best non-metazoan hit [33]. Additional ribosomal protein data were retrieved from the alignments compiled by Hausdorf et al. [18]. All ribosomal protein sequences obtained were aligned by the ClustalW algorithm using default parameters [44]. The resulting ribosomal protein alignments were inspected and adjusted manually for obviously misaligned positions using GeneDoc [45]. Questionably aligned positions were eliminated with GBlocks [46] using less stringent parameters. To test for the effect of missing data on present results [47], we assembled an additional dataset (24 ribosomal proteins, 3,535 amino acids) from which ribosomal protein sequences without acanthocephalan, bdelloid and/or monogonont orthologs were removed.

### Phylogenetic reconstruction

The content of phylogenetic information of the alignments was estimated by the likelihood mapping approach as implemented in Tree-Puzzle 5.2 [48,49], testing all 23,751 possible quartets with exact parameter estimation.

Bayesian inference analyses based on the site-heterogenous CAT model (which allows the amino-acid replacement pattern to vary across a protein alignment; [50]) were performed using PhyloBayes v2.1c [51]. Two independent chains were run simultaneously for 11,210 points each. Chain equilibrium was estimated by plotting the log-likelihood and the alpha parameter as a function of the generation number. The first 500 points were subsequently discarded as burn-in. According to the divergence of bipartition frequencies, both chains reached convergence (maximal difference <0.08, mean difference <0.003), supported by the fact that both chains produced the same consensus tree topology. Taking every 10^th ^sampled tree, a 50% majority rule consensus tree was finally computed using both chains.

ProtTest [52] was used to assess the appropriate model of sequence evolution for maximum likelihood-based tree reconstruction. As ribosomal proteins are likely to evolve similarly, the model was determined for the concatenated dataset, instead of for each single protein. Analyses were then conducted using PhyML [53] and Treefinder [54,55] with the rtREV+I+G+F substitution model [56] and 500 bootstrap replicates. Confidence values for the edges of the maximum likelihood tree (Treefinder) were computed by applying expected likelihood weights (ELWs) [57] to all local rearrangements of tree topology around an edge (1,000 replications). Trees produced in the course of the analysis were further edited using TreeView [58].

To test predefined phylogenetic hypotheses, we used constrained trees and the 'resolve multifurcations' option of Treefinder to obtain the maximum likelihood tree for a specified hypothesis. Thereafter we investigated whether the maximum likelihood trees for these hypotheses are part of the confidence set of trees applying the expected likelihood weights method [57].

## Authors' contributions

AW participated in the design and coordination of the study, carried out the sequence analyses and alignments, participated in the phylogenetic reconstructions and wrote the manuscript. HH provided acanthocephalans, participated in the design of the study and wrote the manuscript. AM participated in the phylogenetic reconstructions. LB and GB provided monogononts. TH conceived the study, participated in its design and coordination and helped to draft the manuscript. All authors read and approved the final manuscript.

## Supplementary Material

Additional file 1 **Supplementary table 1.** List of taxa, number of ribosomal proteins, number of amino acids and the percental coverage per taxon used in our analyses. Click here for file

Additional file 2 **Supplementary table 2.** Complete matrix of taxa and ribosomal proteins used in this analysis with the number of amino acids per ribosomal protein. Maximum length of each ribosomal protein is shown in brackets under each protein name. Click here for file
