# Supplementary material for: EST based phylogenomics of Syndermata questions monophyly of Eurotatoria
Source: BMC Evol Biol. 2008 Dec 29;8:345. doi: 10.1186/1471-2148-8-345 (PMC2654452; doi:10.1186/1471-2148-8-345)
Supplement: Additional file 1 — Supplementary table 1. List of taxa, number of ribosomal proteins, number of amino acids and the percental coverage per taxon used in our analyses. [file 1471-2148-8-345-S1.doc]

| **Species** | **# RP** | **# amino acids** | **% coverage** |
| --- | --- | --- | --- |
| *Anopheles gambiae* | 79 | 11263 | 99,92 |
| *Aplysia californica* | 78 | 10946 | 97,10 |
| *Arenicola marina* | 47 | 7465 | 66,23 |
| *Argopecten irradians* | 77 | 10593 | 93,98 |
| *Ascaris suum* | 78 | 10873 | 96,46 |
| *Barentsia elongata* | 23 | 6246 | 55,41 |
| *Brachionus plicatilis* | 28 | 4255 | 37,75 |
| *Capitella capitata* | 61 | 8872 | 78,71 |
| *Crassostrea spec.* | 77 | 10757 | 95,43 |
| *Daphnia magna* | 79 | 11164 | 99,04 |
| *Echinococcus granulosus* | 76 | 10565 | 93,73 |
| *Echinorhynchus truttae* | 28 | 3204 | 28,42 |
| *Euprymna scolopes* | 62 | 8983 | 79,69 |
| *Flaccisagitta enflata* | 60 | 8122 | 72,05 |
| *Flustra foliacea* | 73 | 10269 | 91,10 |
| *Fugu rubripes* | 79 | 11272 | 100 |
| *Helobdella robusta* | 57 | 7828 | 69,45 |
| *Homo sapiens* | 79 | 11271 | 99,99 |
| *Hydra magnipapillata* | 79 | 11223 | 99,57 |
| *Hypsibius dujardini* | 71 | 9941 | 88,19 |
| *Lumbricus rubellus* | 78 | 11157 | 98,98 |
| *Macrostomum lignano* | 61 | 8137 | 72,19 |
| *Nematostella vectensis* | 68 | 9871 | 87,57 |
| *Philodina roseola* | 72 | 10005 | 88,76 |
| *Pomphorhynchus lavis* | 65 | 7430 | 65,92 |
| *Priapulus caudatus* | 72 | 4053 | 35,96 |
| *Schistosoma spec.* | 79 | 11236 | 99,68 |
| *Spadella cephaloptera* | 65 | 9280 | 82,33 |
| *Xiphinema index* | 74 | 10348 | 91,80 |
