# Supplementary material for: EST based phylogenomics of Syndermata questions monophyly of Eurotatoria
Source: BMC Evol Biol. 2008 Dec 29;8:345. doi: 10.1186/1471-2148-8-345 (PMC2654452; doi:10.1186/1471-2148-8-345)
Supplement: Additional file 2 — Supplementary table 2. Complete matrix of taxa and ribosomal proteins used in this analysis with the number of amino acids per ribosomal protein. Maximum length of each ribosomal protein is shown in brackets under each protein name. [file 1471-2148-8-345-S2.doc]

| **Species** | **L3** (380) | **L4**  (275) | **L5** (248) | **L6** (168) | **L7** (203) | **L7a** (225) | **L8** (248) | **L9** (178) | **L10** (209) | **L10a** (213) |
| --- | --- | --- | --- | --- | --- | --- | --- | --- | --- | --- |
| *Anopheles gambiae* | 380 | 275 | 248 | 167 | 203 | 225 | 248 | 178 | 209 | 213 |
| *Aplysia californica* | 328 | 275 | 187 | 168 | 203 | 225 | 248 | 177 | 209 | 213 |
| *Arenicola marina* | 0 | 38 | 233 | 84 | 202 | 225 | 0 | 178 | 206 | 104 |
| *Argopecten irradians* | 380 | 264 | 248 | 168 | 203 | 225 | 248 | 178 | 209 | 213 |
| *Ascaris suum* | 221 | 271 | 228 | 168 | 202 | 197 | 248 | 175 | 208 | 212 |
| *Barentsia elongata* | 0 | 0 | 248 | 0 | 203 | 225 | 91 | 0 | 209 | 0 |
| *Brachionus plicatilis* | 380 | 0 | 0 | 0 | 203 | 0 | 248 | 0 | 0 | 0 |
| *Capitella capitata* | 379 | 275 | 246 | 147 | 203 | 181 | 220 | 165 | 201 | 213 |
| *Crassostrea spec.* | 380 | 275 | 242 | 168 | 203 | 225 | 205 | 177 | 209 | 213 |
| *Daphnia magna* | 380 | 275 | 248 | 122 | 203 | 225 | 248 | 178 | 209 | 213 |
| *Echinococcus granulosus* | 380 | 275 | 199 | 162 | 105 | 168 | 248 | 178 | 209 | 213 |
| *Echinorhynchus truttae* | 0 | 0 | 0 | 0 | 0 | 218 | 0 | 0 | 0 | 213 |
| *Euprymna scolopes* | 380 | 275 | 248 | 168 | 203 | 224 | 247 | 178 | 209 | 213 |
| *Flaccisagitta enflata* | 194 | 0 | 164 | 149 | 100 | 172 | 210 | 177 | 111 | 196 |
| *Flustra foliacea* | 185 | 0 | 176 | 126 | 171 | 173 | 176 | 177 | 209 | 203 |
| *Fugu rubripes* | 380 | 275 | 248 | 168 | 203 | 225 | 248 | 178 | 209 | 213 |
| *Helobdella robusta* | 375 | 220 | 221 | 126 | 192 | 165 | 205 | 135 | 182 | 213 |
| *Homo sapiens* | 380 | 275 | 248 | 168 | 203 | 225 | 248 | 178 | 209 | 213 |
| *Hydra magnipapillata* | 380 | 275 | 248 | 165 | 203 | 225 | 248 | 177 | 180 | 213 |
| *Hypsibius dujardini* | 0 | 194 | 213 | 0 | 158 | 176 | 248 | 178 | 205 | 188 |
| *Lumbricus rubellus* | 380 | 275 | 248 | 168 | 200 | 225 | 248 | 178 | 209 | 213 |
| *Macrostomum lignano* | 281 | 257 | 248 | 134 | 202 | 0 | 145 | 0 | 171 | 0 |
| *Nematostella vectensis* | 377 | 275 | 248 | 164 | 188 | 208 | 248 | 159 | 201 | 213 |
| *Philodina roseola* | 314 | 234 | 248 | 0 | 201 | 216 | 248 | 178 | 209 | 213 |
| *Pomphorhynchus laevis* | 0 | 0 | 0 | 0 | 121 | 0 | 169 | 154 | 183 | 0 |
| *Priapulus caudatus* | 0 | 56 | 98 | 104 | 0 | 0 | 0 | 150 | 152 | 159 |
| *Schistosoma spec.* | 380 | 275 | 248 | 167 | 202 | 225 | 248 | 177 | 209 | 213 |
| *Spadella cephaloptera* | 380 | 0 | 248 | 168 | 202 | 0 | 0 | 177 | 209 | 213 |
| *Xiphinema index* | 353 | 257 | 200 | 168 | 203 | 225 | 248 | 178 | 209 | 213 |

| **Species** | **L11** (166) | **L12** (163) | **L13** (175) | **L13a** (173) | **L14** (105) | **L15** (204) | **L17** (163) | **L18** (185) | **L18a** (165) | | **L19** (185) |
| --- | --- | --- | --- | --- | --- | --- | --- | --- | --- | --- | --- |
| *Anopheles gambiae* | 166 | 163 | 175 | 173 | 105 | 204 | 163 | 185 | 165 | | 185 |
| *Aplysia californica* | 166 | 163 | 174 | 173 | 105 | 204 | 162 | 185 | 165 | | 185 |
| *Arenicola marina* | 0 | 163 | 116 | 77 | 105 | 0 | 162 | 183 | 165 | | 185 |
| *Argopecten irradians* | 166 | 163 | 175 | 173 | 105 | 0 | 163 | 185 | 165 | | 185 |
| *Ascaris suum* | 166 | 163 | 173 | 172 | 105 | 200 | 163 | 183 | 165 | | 183 |
| *Barentsia elongata* | 166 | 0 | 0 | 173 | 0 | 0 | 160 | 185 | 165 | | 185 |
| *Brachionus plicatilis* | 166 | 163 | 0 | 173 | 0 | 0 | 0 | 0 | 165 | | 0 |
| *Capitella capitata* | 129 | 151 | 175 | 130 | 105 | 204 | 154 | 141 | 160 | | 185 |
| *Crassostrea spec.* | 166 | 163 | 175 | 173 | 105 | 204 | 163 | 185 | 165 | | 185 |
| *Daphnia magna* | 137 | 163 | 174 | 173 | 105 | 204 | 163 | 185 | 165 | | 185 |
| *Echinococcus granulosus* | 166 | 0 | 172 | 173 | 105 | 204 | 162 | 170 | 165 | | 185 |
| *Echinorhynchus truttae* | 166 | 0 | 175 | 0 | 0 | 0 | 0 | 0 | 0 | | 0 |
| *Euprymna scolopes* | 48 | 128 | 175 | 173 | 105 | 0 | 163 | 185 | 165 | | 182 |
| *Flaccisagitta enflata* | 166 | 163 | 129 | 141 | 103 | 177 | 163 | 132 | 162 | | 185 |
| *Flustra foliacea* | 166 | 163 | 160 | 164 | 105 | 204 | 163 | 185 | 165 | | 185 |
| *Fugu rubripes* | 166 | 163 | 175 | 173 | 105 | 204 | 163 | 185 | 165 | | 185 |
| *Helobdella robusta* | 129 | 151 | 174 | 59 | 73 | 204 | 105 | 185 | 103 | | 116 |
| *Homo sapiens* | 166 | 163 | 175 | 173 | 105 | 204 | 163 | 185 | 165 | | 185 |
| *Hydra magnipapillata* | 165 | 163 | 175 | 170 | 105 | 204 | 163 | 185 | 164 | | 185 |
| *Hypsibius dujardini* | 166 | 163 | 133 | 173 | 103 | 204 | 163 | 183 | 165 | | 185 |
| *Lumbricus rubellus* | 166 | 163 | 175 | 173 | 105 | 204 | 163 | 185 | 165 | | 185 |
| *Macrostomum lignano* | 166 | 151 | 175 | 0 | 0 | 132 | 112 | 159 | 160 | | 185 |
| *Nematostella vectensis* | 166 | 124 | 174 | 169 | 105 | 204 | 163 | 171 | 165 | | 154 |
| *Philodina roseola* | 166 | 163 | 0 | 173 | 105 | 204 | 163 | 185 | 165 | | 0 |
| *Pomphorhynchus laevis* | 156 | 163 | 143 | 173 | 0 | 0 | 155 | 155 | 44 | | 176 |
| *Priapulus caudatus* | 0 | 0 | 138 | 0 | 0 | 169 | 0 | 0 | 0 | | 0 |
| *Schistosoma spec.* | 166 | 163 | 175 | 173 | 105 | 204 | 163 | 185 | 165 | | 184 |
| *Spadella cephaloptera* | 166 | 0 | 174 | 173 | 103 | 204 | 163 | 185 | 165 | | 0 |
| *Xiphinema index* | 166 | 163 | 174 | 173 | 105 | 204 | 163 | 185 | 165 | | 185 |
|  |  |  |  |  |  |  |  |  | |  |  |
|  |  |  |  |  |  |  |  |  | |  |  |
| **Species** | **L21** (156) | **L22** (96) | **L23** (139) | **L23a** (135) | **L24** (117) | **L26** (138) | **L27** (136) | **L27a**  (136) | | **L28** (63) | **L29** (50) |
| *Anopheles gambiae* | 155 | 96 | 139 | 135 | 117 | 138 | 135 | 136 | | 62 | 50 |
| *Aplysia californica* | 156 | 96 | 139 | 135 | 0 | 138 | 136 | 135 | | 63 | 50 |
| *Arenicola marina* | 156 | 96 | 139 | 134 | 117 | 136 | 136 | 110 | | 63 | 0 |
| *Argopecten irradians* | 156 | 96 | 139 | 135 | 117 | 137 | 136 | 136 | | 63 | 0 |
| *Ascaris suum* | 153 | 95 | 139 | 135 | 116 | 133 | 136 | 136 | | 63 | 50 |
| *Barentsia elongata* | 150 | 65 | 0 | 0 | 0 | 0 | 136 | 78 | | 0 | 50 |
| *Brachionus plicatilis* | 106 | 92 | 138 | 127 | 0 | 0 | 136 | 0 | | 0 | 0 |
| *Capitella capitata* | 156 | 36 | 64 | 107 | 75 | 104 | 0 | 112 | | 0 | 0 |
| *Crassostrea spec.* | 156 | 96 | 138 | 135 | 117 | 137 | 136 | 136 | | 33 | 50 |
| *Daphnia magna* | 156 | 96 | 139 | 135 | 117 | 138 | 136 | 136 | | 63 | 50 |
| *Echinococcus granulosus* | 156 | 96 | 139 | 135 | 117 | 138 | 134 | 136 | | 63 | 50 |
| *Echinorhynchus truttae* | 152 | 0 | 0 | 135 | 0 | 0 | 0 | 0 | | 0 | 0 |
| *Euprymna scolopes* | 156 | 0 | 134 | 135 | 117 | 137 | 136 | 135 | | 63 | 0 |
| *Flaccisagitta enflata* | 155 | 96 | 139 | 113 | 117 | 138 | 0 | 136 | | 0 | 0 |
| *Flustra foliacea* | 156 | 96 | 139 | 135 | 117 | 138 | 136 | 73 | | 63 | 50 |
| *Fugu rubripes* | 156 | 96 | 139 | 135 | 117 | 137 | 136 | 136 | | 63 | 50 |
| *Helobdella robusta* | 127 | 71 | 139 | 129 | 75 | 105 | 0 | 135 | | 0 | 0 |
| *Homo sapiens* | 156 | 95 | 139 | 135 | 117 | 137 | 136 | 136 | | 63 | 50 |
| *Hydra magnipapillata* | 155 | 94 | 139 | 135 | 117 | 136 | 136 | 136 | | 62 | 50 |
| *Hypsibius dujardini* | 156 | 57 | 119 | 119 | 117 | 138 | 136 | 136 | | 63 | 50 |
| *Lumbricus rubellus* | 156 | 92 | 139 | 135 | 117 | 137 | 136 | 136 | | 63 | 50 |
| *Macrostomum lignano* | 156 | 96 | 135 | 135 | 0 | 138 | 135 | 0 | | 63 | 0 |
| *Nematostella vectensis* | 155 | 36 | 139 | 135 | 117 | 136 | 123 | 136 | | 0 | 0 |
| *Philodina roseola* | 156 | 0 | 115 | 135 | 117 | 136 | 136 | 134 | | 63 | 50 |
| *Pomphorhynchus laevis* | 152 | 95 | 138 | 0 | 117 | 136 | 136 | 135 | | 60 | 48 |
| *Priapulus caudatus* | 0 | 96 | 132 | 116 | 117 | 0 | 136 | 133 | | 0 | 0 |
| *Schistosoma spec.* | 139 | 96 | 139 | 135 | 117 | 138 | 134 | 136 | | 63 | 50 |
| *Spadella cephaloptera* | 156 | 96 | 139 | 0 | 117 | 138 | 136 | 136 | | 62 | 50 |
| *Xiphinema index* | 155 | 95 | 139 | 0 | 117 | 137 | 134 | 136 | | 63 | 50 |

| **Species** | **L30** (104) | **L31** (109) | **L32** (130) | **L34** (99) | **L35** (122) | | **L35a** (101) | | **L36** (71) | | **L36a** (101) | | | **L37** (85) | | **L37a** (88) |
| --- | --- | --- | --- | --- | --- | --- | --- | --- | --- | --- | --- | --- | --- | --- | --- | --- |
| *Anopheles gambiae* | 104 | 109 | 130 | 99 | 122 | | 101 | | 71 | | 101 | | | 85 | | 88 |
| *Aplysia californica* | 104 | 109 | 130 | 99 | 121 | | 99 | | 71 | | 101 | | | 84 | | 88 |
| *Arenicola marina* | 104 | 109 | 123 | 99 | 122 | | 101 | | 68 | | 101 | | | 85 | | 88 |
| *Argopecten irradians* | 104 | 109 | 130 | 0 | 121 | | 101 | | 71 | | 100 | | | 85 | | 0 |
| *Ascaris suum* | 104 | 108 | 79 | 96 | 120 | | 100 | | 51 | | 99 | | | 82 | | 88 |
| *Barentsia elongata* | 68 | 109 | 130 | 0 | 0 | | 0 | | 0 | | 101 | | | 0 | | 88 |
| *Brachionus plicatilis* | 0 | 0 | 130 | 0 | 0 | | 0 | | 0 | | 0 | | | 0 | | 88 |
| *Capitella capitata* | 102 | 81 | 128 | 99 | 75 | | 51 | | 0 | | 43 | | | 47 | | 87 |
| *Crassostrea spec.* | 104 | 107 | 127 | 99 | 122 | | 101 | | 0 | | 100 | | | 47 | | 87 |
| *Daphnia magna* | 104 | 109 | 130 | 99 | 122 | | 101 | | 71 | | 101 | | | 81 | | 88 |
| *Echinococcus granulosus* | 0 | 109 | 130 | 99 | 121 | | 101 | | 70 | | 101 | | | 83 | | 0 |
| *Echinorhynchus truttae* | 101 | 0 | 0 | 0 | 0 | | 101 | | 0 | | 0 | | | 85 | | 0 |
| *Euprymna scolopes* | 45 | 109 | 130 | 99 | 122 | | 101 | | 0 | | 0 | | | 0 | | 0 |
| *Flaccisagitta enflata* | 104 | 109 | 130 | 99 | 122 | | 100 | | 0 | | 101 | | | 85 | | 88 |
| *Flustra foliacea* | 104 | 108 | 130 | 99 | 122 | | 101 | | 71 | | 101 | | | 85 | | 88 |
| *Fugu rubripes* | 104 | 109 | 130 | 99 | 122 | | 99 | | 71 | | 101 | | | 85 | | 88 |
| *Helobdella robusta* | 96 | 64 | 52 | 0 | 70 | | 97 | | 0 | | 0 | | | 84 | | 43 |
| *Homo sapiens* | 104 | 109 | 130 | 99 | 122 | | 99 | | 71 | | 101 | | | 85 | | 88 |
| *Hydra magnipapillata* | 104 | 109 | 130 | 99 | 122 | | 101 | | 70 | | 101 | | | 85 | | 88 |
| *Hypsibius dujardini* | 104 | 109 | 130 | 99 | 120 | | 101 | | 71 | | 101 | | | 85 | | 88 |
| *Lumbricus rubellus* | 104 | 109 | 130 | 99 | 122 | | 101 | | 71 | | 101 | | | 52 | | 88 |
| *Macrostomum lignano* | 0 | 109 | 130 | 0 | 122 | | 100 | | 0 | | 0 | | | 0 | | 88 |
| *Nematostella vectensis* | 104 | 109 | 90 | 99 | 121 | | 101 | | 0 | | 95 | | | 82 | | 33 |
| *Philodina roseola* | 63 | 109 | 130 | 99 | 122 | | 101 | | 71 | | 0 | | | 85 | | 88 |
| *Pomphorhynchus laevis* | 103 | 101 | 130 | 98 | 0 | | 101 | | 70 | | 0 | | | 85 | | 88 |
| *Priapulus caudatus* | 0 | 0 | 0 | 0 | 88 | | 101 | | 71 | | 0 | | | 85 | | 88 |
| *Schistosoma spec.* | 104 | 109 | 130 | 99 | 122 | | 101 | | 70 | | 101 | | | 83 | | 88 |
| *Spadella cephaloptera* | 104 | 109 | 130 | 99 | 122 | | 100 | | 71 | | 101 | | | 84 | | 88 |
| *Xiphinema index* | 0 | 109 | 126 | 0 | 122 | | 101 | | 0 | | 101 | | | 85 | | 0 |
| **Species** | **L38** (61) | **L39** (51) | **L40** (52) | **L41** (18) | **P0** (272) | **P1** (76) | | **P2** (79) | | **S2** (225) | | **S3** (219) | **S3a** (247) | |  | |
| *Anopheles gambiae* | 61 | 51 | 52 | 18 | 272 | 76 | | 79 | | 225 | | 219 | 247 | |  | |
| *Aplysia californica* | 61 | 51 | 52 | 18 | 272 | 75 | | 79 | | 163 | | 219 | 247 | |  | |
| *Arenicola marina* | 0 | 51 | 52 | 0 | 0 | 76 | | 69 | | 192 | | 0 | 183 | |  | |
| *Argopecten irradians* | 0 | 0 | 52 | 18 | 272 | 75 | | 79 | | 225 | | 219 | 243 | |  | |
| *Ascaris suum* | 59 | 51 | 51 | 0 | 266 | 76 | | 78 | | 224 | | 217 | 241 | |  | |
| *Barentsia elongata* | 0 | 51 | 52 | 0 | 0 | 76 | | 79 | | 224 | | 0 | 247 | |  | |
| *Brachionus plicatilis* | 61 | 47 | 0 | 0 | 0 | 0 | | 0 | | 0 | | 0 | 0 | |  | |
| *Capitella capitata* | 55 | 0 | 0 | 0 | 222 | 61 | | 69 | | 225 | | 215 | 175 | |  | |
| *Crassostrea spec.* | 38 | 49 | 52 | 18 | 227 | 74 | | 79 | | 225 | | 219 | 247 | |  | |
| *Daphnia magna* | 61 | 51 | 52 | 18 | 272 | 75 | | 79 | | 225 | | 208 | 247 | |  | |
| *Echinococcus granulosus* | 61 | 51 | 50 | 0 | 263 | 76 | | 79 | | 225 | | 218 | 235 | |  | |
| *Echinorhynchus truttae* | 0 | 51 | 0 | 18 | 0 | 0 | | 0 | | 0 | | 219 | 244 | |  | |
| *Euprymna scolopes* | 0 | 0 | 52 | 0 | 249 | 73 | | 63 | | 220 | | 219 | 247 | |  | |
| *Flaccisagitta enflata* | 61 | 51 | 52 | 0 | 156 | 0 | | 0 | | 162 | | 202 | 186 | |  | |
| *Flustra foliacea* | 61 | 51 | 52 | 0 | 272 | 75 | | 78 | | 188 | | 219 | 247 | |  | |
| *Fugu rubripes* | 61 | 51 | 52 | 18 | 272 | 75 | | 79 | | 225 | | 219 | 247 | |  | |
| *Helobdella robusta* | 35 | 51 | 0 | 0 | 141 | 0 | | 57 | | 188 | | 215 | 133 | |  | |
| *Homo sapiens* | 61 | 51 | 52 | 18 | 272 | 75 | | 79 | | 225 | | 219 | 247 | |  | |
| *Hydra magnipapillata* | 61 | 51 | 52 | 18 | 272 | 75 | | 79 | | 225 | | 218 | 246 | |  | |
| *Hypsibius dujardini* | 61 | 51 | 52 | 18 | 193 | 76 | | 79 | | 0 | | 219 | 247 | |  | |
| *Lumbricus rubellus* | 61 | 51 | 52 | 18 | 272 | 0 | | 79 | | 225 | | 219 | 247 | |  | |
| *Macrostomum lignano* | 0 | 0 | 52 | 0 | 255 | 58 | | 72 | | 213 | | 219 | 240 | |  | |
| *Nematostella vectensis* | 35 | 49 | 52 | 0 | 223 | 76 | | 79 | | 149 | | 215 | 239 | |  | |
| *Philodina roseola* | 61 | 51 | 51 | 0 | 264 | 57 | | 79 | | 225 | | 219 | 247 | |  | |
| *Pomphorhynchus laevis* | 61 | 51 | 51 | 18 | 151 | 76 | | 79 | | 164 | | 0 | 181 | |  | |
| *Priapulus caudatus* | 0 | 51 | 52 | 18 | 0 | 0 | | 0 | | 0 | | 0 | 136 | |  | |
| *Schistosoma spec.* | 61 | 51 | 52 | 18 | 272 | 75 | | 79 | | 225 | | 219 | 246 | |  | |
| *Spadella cephaloptera* | 61 | 51 | 52 | 0 | 272 | 76 | | 78 | | 133 | | 219 | 0 | |  | |
| *Xiphinema index* | 33 | 51 | 52 | 0 | 272 | 0 | | 79 | | 216 | | 219 | 247 | |  | |

| **Species** | **S4** (257) | **S5** (187) | | **S6** (203) | | **S7** (164) | | **S8** (205) | | **S9** (170) | **S10** (98) | | **S11** (137) | | **S12** (116) | | **S13** (151) | |  | |
| --- | --- | --- | --- | --- | --- | --- | --- | --- | --- | --- | --- | --- | --- | --- | --- | --- | --- | --- | --- | --- |
| *Anopheles gambiae* | 257 | 187 | | 203 | | 164 | | 203 | | 170 | 98 | | 134 | | 116 | | 151 | |  | |
| *Aplysia californica* | 257 | 186 | | 203 | | 163 | | 205 | | 170 | 97 | | 137 | | 116 | | 151 | |  | |
| *Arenicola marina* | 256 | 0 | | 195 | | 164 | | 94 | | 0 | 34 | | 0 | | 0 | | 151 | |  | |
| *Argopecten irradians* | 257 | 135 | | 203 | | 164 | | 205 | | 170 | 98 | | 137 | | 116 | | 151 | |  | |
| *Ascaris suum* | 247 | 187 | | 202 | | 161 | | 203 | | 166 | 97 | | 137 | | 115 | | 151 | |  | |
| *Barentsia elongata* | 257 | 0 | | 0 | | 0 | | 0 | | 170 | 98 | | 137 | | 0 | | 151 | |  | |
| *Brachionus plicatilis* | 257 | 0 | | 0 | | 164 | | 200 | | 0 | 0 | | 135 | | 116 | | 0 | |  | |
| *Capitella capitata* | 257 | 165 | | 202 | | 0 | | 90 | | 0 | 83 | | 106 | | 116 | | 132 | |  | |
| *Crassostrea spec.* | 257 | 187 | | 203 | | 35 | | 205 | | 170 | 98 | | 137 | | 116 | | 150 | |  | |
| *Daphnia magna* | 257 | 187 | | 203 | | 164 | | 203 | | 167 | 96 | | 137 | | 116 | | 151 | |  | |
| *Echinococcus granulosus* | 257 | 186 | | 202 | | 164 | | 204 | | 168 | 95 | | 136 | | 113 | | 151 | |  | |
| *Echinorhynchus truttae* | 113 | 0 | | 0 | | 160 | | 201 | | 0 | 0 | | 0 | | 0 | | 0 | |  | |
| *Euprymna scolopes* | 248 | 187 | | 101 | | 0 | | 205 | | 0 | 92 | | 0 | | 0 | | 151 | |  | |
| *Flaccisagitta enflata* | 195 | 0 | | 175 | | 0 | | 0 | | 0 | 98 | | 137 | | 116 | | 151 | |  | |
| *Flustra foliacea* | 212 | 187 | | 203 | | 164 | | 205 | | 170 | 98 | | 124 | | 116 | | 151 | |  | |
| *Fugu rubripes* | 257 | 187 | | 203 | | 164 | | 205 | | 170 | 98 | | 137 | | 116 | | 151 | |  | |
| *Helobdella robusta* | 232 | 165 | | 202 | | 0 | | 161 | | 0 | 97 | | 107 | | 101 | | 116 | |  | |
| *Homo sapiens* | 257 | 187 | | 203 | | 164 | | 205 | | 170 | 98 | | 137 | | 116 | | 151 | |  | |
| *Hydra magnipapillata* | 257 | 187 | | 203 | | 162 | | 205 | | 170 | 96 | | 137 | | 116 | | 151 | |  | |
| *Hypsibius dujardini* | 183 | 167 | | 203 | | 158 | | 202 | | 170 | 98 | | 137 | | 116 | | 151 | |  | |
| *Lumbricus rubellus* | 257 | 187 | | 203 | | 164 | | 205 | | 170 | 98 | | 136 | | 116 | | 151 | |  | |
| *Macrostomum lignano* | 213 | 187 | | 197 | | 91 | | 186 | | 170 | 0 | | 0 | | 115 | | 151 | |  | |
| *Nematostella vectensis* | 199 | 140 | | 203 | | 163 | | 143 | | 170 | 96 | | 69 | | 101 | | 91 | |  | |
| *Philodina roseola* | 257 | 186 | | 203 | | 163 | | 196 | | 170 | 98 | | 137 | | 116 | | 151 | |  | |
| *Pomphorhynchus laevis* | 137 | 129 | | 0 | | 126 | | 146 | | 167 | 97 | | 0 | | 112 | | 151 | |  | |
| *Priapulus caudatus* | 0 | 0 | | 0 | | 0 | | 159 | | 143 | 96 | | 137 | | 116 | | 0 | |  | |
| *Schistosoma spec.* | 257 | 187 | | 203 | | 164 | | 204 | | 0 | 97 | | 136 | | 116 | | 151 | |  | |
| *Spadella cephaloptera* | 215 | 0 | | 191 | | 164 | | 205 | | 170 | 0 | | 137 | | 116 | | 151 | |  | |
| *Xiphinema index* | 257 | 187 | | 201 | | 164 | | 205 | | 170 | 98 | | 137 | | 116 | | 151 | |  | |
| **Species** | **S14** (148) | | **S15** (135) | | **S15a** (130) | | **S16** (138) | | **S17** (101) | | | **S18** (152) | | **S19** (130) | | **S20** (101) | | **S21** (78) | | **S23** (141) |
| *Anopheles gambiae* | 148 | | 135 | | 130 | | 138 | | 101 | | | 152 | | 129 | | 101 | | 78 | | 141 |
| *Aplysia californica* | 147 | | 134 | | 126 | | 138 | | 91 | | | 151 | | 130 | | 100 | | 78 | | 141 |
| *Arenicola marina* | 0 | | 135 | | 130 | | 138 | | 101 | | | 114 | | 130 | | 0 | | 78 | | 141 |
| *Argopecten irradians* | 147 | | 135 | | 130 | | 138 | | 101 | | | 152 | | 130 | | 101 | | 78 | | 140 |
| *Ascaris suum* | 146 | | 133 | | 128 | | 137 | | 100 | | | 149 | | 130 | | 101 | | 76 | | 139 |
| *Barentsia elongata* | 97 | | 135 | | 130 | | 138 | | 101 | | | 152 | | 130 | | 101 | | 78 | | 141 |
| *Brachionus plicatilis* | 148 | | 135 | | 130 | | 0 | | 0 | | | 148 | | 0 | | 101 | | 0 | | 0 |
| *Capitella capitata* | 126 | | 101 | | 130 | | 130 | | 100 | | | 127 | | 130 | | 101 | | 0 | | 141 |
| *Crassostrea spec.* | 109 | | 135 | | 130 | | 138 | | 101 | | | 152 | | 130 | | 101 | | 78 | | 140 |
| *Daphnia magna* | 148 | | 135 | | 130 | | 138 | | 101 | | | 152 | | 130 | | 101 | | 78 | | 141 |
| *Echinococcus granulosus* | 148 | | 135 | | 130 | | 136 | | 101 | | | 152 | | 129 | | 97 | | 78 | | 141 |
| *Echinorhynchus truttae* | 0 | | 135 | | 130 | | 138 | | 0 | | | 150 | | 0 | | 0 | | 0 | | 0 |
| *Euprymna scolopes* | 148 | | 135 | | 0 | | 138 | | 0 | | | 152 | | 130 | | 84 | | 0 | | 141 |
| *Flaccisagitta enflata* | 148 | | 134 | | 130 | | 138 | | 101 | | | 0 | | 130 | | 101 | | 0 | | 141 |
| *Flustra foliacea* | 147 | | 135 | | 130 | | 138 | | 101 | | | 152 | | 129 | | 101 | | 78 | | 141 |
| *Fugu rubripes* | 148 | | 135 | | 130 | | 138 | | 101 | | | 152 | | 130 | | 101 | | 78 | | 141 |
| *Helobdella robusta* | 147 | | 116 | | 104 | | 47 | | 37 | | | 65 | | 113 | | 100 | | 0 | | 140 |
| *Homo sapiens* | 148 | | 135 | | 130 | | 138 | | 101 | | | 152 | | 130 | | 101 | | 78 | | 141 |
| *Hydra magnipapillata* | 148 | | 135 | | 130 | | 138 | | 101 | | | 152 | | 130 | | 101 | | 77 | | 141 |
| *Hypsibius dujardini* | 148 | | 135 | | 130 | | 138 | | 101 | | | 151 | | 130 | | 99 | | 77 | | 141 |
| *Lumbricus rubellus* | 147 | | 135 | | 130 | | 138 | | 101 | | | 152 | | 130 | | 101 | | 78 | | 141 |
| *Macrostomum lignano* | 145 | | 135 | | 0 | | 0 | | 97 | | | 152 | | 129 | | 101 | | 69 | | 140 |
| *Nematostella vectensis* | 98 | | 135 | | 129 | | 112 | | 53 | | | 64 | | 130 | | 84 | | 0 | | 141 |
| *Philodina roseola* | 148 | | 135 | | 130 | | 138 | | 101 | | | 150 | | 127 | | 80 | | 74 | | 141 |
| *Pomphorhynchus laevis* | 147 | | 135 | | 130 | | 138 | | 101 | | | 78 | | 129 | | 101 | | 78 | | 140 |
| *Priapulus caudatus* | 148 | | 0 | | 0 | | 0 | | 96 | | | 146 | | 0 | | 101 | | 0 | | 64 |
| *Schistosoma spec.* | 148 | | 135 | | 130 | | 138 | | 101 | | | 152 | | 129 | | 101 | | 78 | | 141 |
| *Spadella cephaloptera* | 148 | | 135 | | 130 | | 138 | | 101 | | | 151 | | 130 | | 101 | | 78 | | 141 |
| *Xiphinema index* | 148 | | 135 | | 72 | | 138 | | 101 | | | 152 | | 130 | | 0 | | 77 | | 141 |

| **Species** | **S24** (113) | **S25** (88) | **S26** (97) | **S27** (82) | **S27a** (70) | **S28** (56) | **S29** (54) | **S30** (59) | **SA** (208) |
| --- | --- | --- | --- | --- | --- | --- | --- | --- | --- |
| *Anopheles gambiae* | 112 | 88 | 97 | 82 | 70 | 56 | 54 | 58 | 207 |
| *Aplysia californica* | 113 | 88 | 94 | 82 | 69 | 56 | 54 | 59 | 205 |
| *Arenicola marina* | 113 | 88 | 0 | 82 | 70 | 56 | 0 | 59 | 0 |
| *Argopecten irradians* | 113 | 87 | 97 | 82 | 70 | 0 | 54 | 59 | 208 |
| *Ascaris suum* | 113 | 87 | 95 | 82 | 69 | 56 | 54 | 58 | 207 |
| *Barentsia elongata* | 91 | 88 | 0 | 82 | 70 | 0 | 0 | 0 | 185 |
| *Brachionus plicatilis* | 109 | 0 | 0 | 0 | 0 | 0 | 0 | 0 | 208 |
| *Capitella capitata* | 0 | 88 | 96 | 74 | 0 | 56 | 0 | 0 | 208 |
| *Crassostrea spec.* | 112 | 88 | 97 | 82 | 46 | 56 | 0 | 59 | 208 |
| *Daphnia magna* | 113 | 88 | 97 | 82 | 70 | 56 | 54 | 46 | 208 |
| *Echinococcus granulosus* | 113 | 88 | 96 | 80 | 70 | 0 | 54 | 59 | 207 |
| *Echinorhynchus truttae* | 0 | 0 | 0 | 0 | 0 | 56 | 0 | 52 | 204 |
| *Euprymna scolopes* | 113 | 0 | 0 | 0 | 70 | 56 | 0 | 59 | 186 |
| *Flaccisagitta enflata* | 0 | 88 | 0 | 82 | 44 | 55 | 54 | 0 | 208 |
| *Flustra foliacea* | 113 | 88 | 97 | 82 | 70 | 55 | 54 | 59 | 158 |
| *Fugu rubripes* | 113 | 88 | 97 | 82 | 70 | 56 | 54 | 59 | 208 |
| *Helobdella robusta* | 0 | 0 | 96 | 37 | 0 | 0 | 0 | 0 | 205 |
| *Homo sapiens* | 113 | 88 | 97 | 82 | 70 | 56 | 54 | 59 | 208 |
| *Hydra magnipapillata* | 113 | 88 | 97 | 82 | 70 | 56 | 54 | 59 | 208 |
| *Hypsibius dujardini* | 113 | 88 | 96 | 82 | 70 | 55 | 54 | 59 | 196 |
| *Lumbricus rubellus* | 113 | 88 | 97 | 82 | 70 | 56 | 54 | 59 | 208 |
| *Macrostomum lignano* | 108 | 0 | 94 | 81 | 70 | 0 | 54 | 59 | 208 |
| *Nematostella vectensis* | 113 | 81 | 97 | 80 | 70 | 38 | 0 | 59 | 208 |
| *Philodina roseola* | 113 | 88 | 97 | 82 | 70 | 56 | 54 | 57 | 0 |
| *Pomphorhynchus laevis* | 113 | 87 | 95 | 80 | 69 | 54 | 49 | 50 | 174 |
| *Priapulus caudatus* | 113 | 0 | 97 | 82 | 0 | 56 | 53 | 0 | 0 |
| *Schistosoma spec.* | 108 | 88 | 97 | 82 | 70 | 56 | 54 | 59 | 205 |
| *Spadella cephaloptera* | 113 | 88 | 97 | 82 | 70 | 56 | 54 | 0 | 208 |
| *Xiphinema index* | 112 | 54 | 97 | 82 | 70 | 56 | 54 | 59 | 208 |
